# Angiogenesis-Related Pathways in the Pathogenesis of Ovarian Cancer

**DOI:** 10.3390/ijms140815885

**Published:** 2013-07-30

**Authors:** Nikos G. Gavalas, Michalis Liontos, Sofia-Paraskevi Trachana, Tina Bagratuni, Calliope Arapinis, Christine Liacos, Meletios A. Dimopoulos, Aristotle Bamias

**Affiliations:** Department of Clinical Therapeutics, Medical School, University of Athens, Alexandra Hospital, 80 Vas. Sofias Avenue, Athens 115 28, Greece; E-Mails: ngavalas@med.uoa.gr (N.G.G.); mliontos@gmail.com (L.M.); sp.voula@yahoo.com (S.-P.T.); tbagratuni@hotmail.co.uk (T.B.); karapini@gmail.com (C.A.); liakou@med.uoa.gr (C.L.); mdimop@med.uoa.gr (M.A.G.)

**Keywords:** ovarian, cancer, angiogenesis, pathway, VEGF, PDGF, FGF, Ang, Tie2

## Abstract

Ovarian Cancer represents the most fatal type of gynecological malignancies. A number of processes are involved in the pathogenesis of ovarian cancer, especially within the tumor microenvironment. Angiogenesis represents a hallmark phenomenon in cancer, and it is responsible for tumor spread and metastasis in ovarian cancer, among other tumor types, as it leads to new blood vessel formation. In recent years angiogenesis has been given considerable attention in order to identify targets for developing effective anti-tumor therapies. Growth factors have been identified to play key roles in driving angiogenesis and, thus, the formation of new blood vessels that assist in “feeding” cancer. Such molecules include the vascular endothelial growth factor (VEGF), the platelet derived growth factor (PDGF), the fibroblast growth factor (FGF), and the angiopoietin/Tie2 receptor complex. These proteins are key players in complex molecular pathways within the tumor cell and they have been in the spotlight of the development of anti-angiogenic molecules that may act as stand-alone therapeutics, or in concert with standard treatment regimes such as chemotherapy. The pathways involved in angiogenesis and molecules that have been developed in order to combat angiogenesis are described in this paper.

## 1. Ovarian Cancer: Pathogenesis and Clinical Aspects

Cancer is a major public health problem and it constitutes one of the most frequent causes of death in the Western world. A total of 1,638,910 new cancer cases, and 577,190 deaths from cancer, were estimated to occur in the United States in 2012 [1]. The economic burden of cancer is associated with expenditures including aspects such as prevention, screening and treatment services, and the lost productivity due to cancer-related death [2]. The five-year net burden for the American Health System has reached almost $21 billion, whereas later findings calculate the total cost of cancer reaching up to $1 trillion in 2009.

Ovarian cancer is the most fatal among gynecologic cancers. In terms of pathology, epithelial ovarian cancers are classified into five main types including High-Grade Serous Carcinomas (HGSC), Endometroid Carcinomas (EC), Clear Cell Carcinomas (CCC), Mucinous Carcinomas (MC), and Low-Grade Serous Carcinomas (LGSC) [3]. These distinct histological subtypes share few molecular similarities and many of them arise from non-ovarian tissues [4]. Interestingly, contemporary therapeutic approaches are common for all subtypes of epithelial ovarian carcinomas, while the effectiveness of the cytotoxic drugs used has reached a plateau, as indicated by the unaltered five-year survival of ovarian cancer patients the last 15 years [5]. Ovarian carcinomas have been considered to arise from the epithelium that lines the ovarian surface. The latter is composed from a layer of flat to cuboidal epithelial cells that derive from the embryonic coleomic epithelium [6].

Invagination of the ovarian surface epithelium during ovulatory cycles forms inclusion cysts. The exposure of invaginated epithelium to hormonal stimulation accounts for its metaplasia and promotes its malignant transformation [7]. The normal ovary, though, lacks constituents that resemble the major histological subtypes of ovarian carcinoma. Furthermore, ovaries develop embryologically from mesodermal epithelium on the urogenital ridge, separate from the müllerian ducts, and although inclusion cysts are frequently encountered in ovaries, there is no histological evidence that these structures could constitute the precursors of high-grade serous carcinomas.

An alternative theory proposed by Lauchlan [8,9] suggests that tumors with a müllerian phenotype arise from müllerian-type tissue, outside from the primary Müllerian system, that is collectively referred as the “secondary Müllerian system”. This includes the Müllerian type epithelium encountered in ovarian inclusion cysts. As these tumors enlarge, they obliterate ovarian tissue resulting in an adnexal tumor that appears to have arisen from the ovary. This theory explains the pathogenesis of some tumors, identical to ovarian carcinomas that develop, despite the ovaries or the ovaries along with the fallopian tubes and the uterus having been previously removed. However, the value of this theory is limited since rarely, if ever, have [10] premalignant lesions resembling ovarian carcinomas in paratubal or paraovarian cysts been recognized [4].

During the last decade, the interest in the pathogenesis of ovarian cancer, and especially High Grade Serous Carcinomas, has been transferred to the fallopian tube. More specifically, in 2001 Pick *et al.* described dysplastic lesions in the fallopian tube of women, with germline BRCA1 mutation, that were subjected to prophylactic salpingoophorectomy [11]. These lesions, later characterized as Serous Tubular Intraepithelial Carcinomas (STICs), were also described in a number of subsequent studies [10,12–14]. Based on this observation, Kindelberger *et al.* not only recognized STICs after careful examination of the fimbria in a series of serous ovarian carcinomas, but also identified identical *TP53* mutations among STICs and the corresponding invasive carcinomas [15], thus providing the etiological link between these two entities.

The heterogenous group of epithelial ovarian carcinomas is reflected, not only in histopathology, but also in genetic lesions. Based on morphological and genetic analysis, Shih and Kurman have proposed the dualistic model for ovarian carcinogenesis [16]. They have classified epithelial ovarian carcinomas, based on the genetic alterations implicated in their carcinogenesis, into two types.

Type I tumors include low-grade serous carcinomas, mucinous carcinomas, clear cell carcinomas, endometroid carcinomas, and malignant Brenner tumors [16]. They are slow growing tumors and are usually detected at a low FIGO stage, with most of these tumors confined in the ovary [17]. Their development proceeds in a stepwise fashion from well-recognized precursor lesions and are genetically stable [16]. Mutations of genes such as *KRAS* and *ERBB2* that deregulate MAPK signaling pathway drive carcinogenesis in approximately 70% of LSGC [18,19]. In Low Grade Endometroid and Clear Cell Carcinomas similar genetic alterations are detected, such as those that affect the PI3K signaling pathway. These include activating mulation of the *PIK3CA* in approximately 50% of cases [20]. Genome-wide mutation analysis in these tumors has also highlighted the implication of tumor suppressor genes in their pathogenesis [21].

In sharp contrast to type I ovarian tumors, the predominant genetic alteration that drives carcinogenesis in type II tumors are *TP53* mutations. High Grade Serous Carcinomas harbor *TP53* mutations in >95% of cases [22], and analogous is the percentage in High-Grade Endometroid Carcinomas, an entity that is often morphologically indistinguishable from serous counterparts. As anticipated, mutations in the “guardian of the genome” *TP53* gene, results in increased genomic instability detected in HGSCs [23,24]. DNA copy number gains or losses have been frequently detected in genes such as *PIK3CA* [25,26]. Mutations in *BRCA1/2* genes that characterize cases of familiar ovarian carcinomas are rarely encountered in sporadic cases.

Even newly approved molecular therapies for ovarian carcinomas [27–29], despite being promising, lack well defined biomarkers that could improve their effective use. The above, underscore the need to improve our understanding of ovarian cancer at the molecular and cellular level by recognizing the cell of origin, identifying precancerous lesions, and delineating the pathogenesis of the disease.

One of the main targets for future drug developments is angiogenesis.

## 2. Angiogenesis in Cancer Pathogenesis

Angiogenesis refers to the process of the formation of new vessels, and it constitutes a hallmark process of cancer progression and metastasis. The angiogenetic process is rather complex and involves a large number of cytokines and associated receptors. It occurs during the menstrual cycle, and also wound healing in the ovaries and the endometrium, in adult life. The angiogenesis term was founded over a century ago [30], but its meaning was not fully elucidated until the 1960s decade when Judah Folkman discovered that tiny tumors grew to about 1–2 mm in size and also stopped expanding in the absence of the vascularization process [31].

Angiogenesis has been shown to be a necessary process for oncogenesis, as well as subsequent tumor growth and dissemination through metastases.

Microvessel density related quantitative analysis in a number of different cancer types in patients revealed that the angiogenic switch and the initiation of angiogenesis also occur during the growth of human cancers [32,33]. In ovarian cancer, angiogenesis has also been associated with the formation of malignant ascites [34,35].

During oncogenesis, tumor endothelial cells, which line blood vessels, may divide up to 50 times faster than normal endothelial cells, providing them with a significant growth advantage over their normal counterparts. Continuous neovascularisation allows tumor cells to grow beyond a diffusion-limited size, therefore rendering angiogenesis an important process in the pathogenesis of cancer. The architecture of the tumor blood vessels exhibits differences to the architecture of normal blood vessels, more specifically, abnormalities. Tumor vessels exhibit high vascular permeability, poor blood flow, and a rather irregular shape when compared to normal ones [36]. The elimination of the angiogenic process may result in the inability of the tumor to grow further although cell proliferation occurs, counter balanced by apoptosis, as observed in tumor dormant areas, and it allows metastasis to occur [37].

The mechanism by which angiogenesis occurs is quite complex and it is yet to be elucidated although advances in this field of research are quite intensive. Cancer cells release pro-angiogenic factors, such as the Vascular Endothelial Growth Factor (VEGF) and the platelet derived growth factor (PDGF) [38–40]. These factors act by activating endothelial cells, thus leading to new blood vessel formation, in order to initiate angiogenesis. The angiogenic process itself in the tumor microenvironment involves the interplay of angiogenic growth factors with their corresponding receptors, leading to endothelial cell activation, and also vascular remodeling.

**Figure 1:**
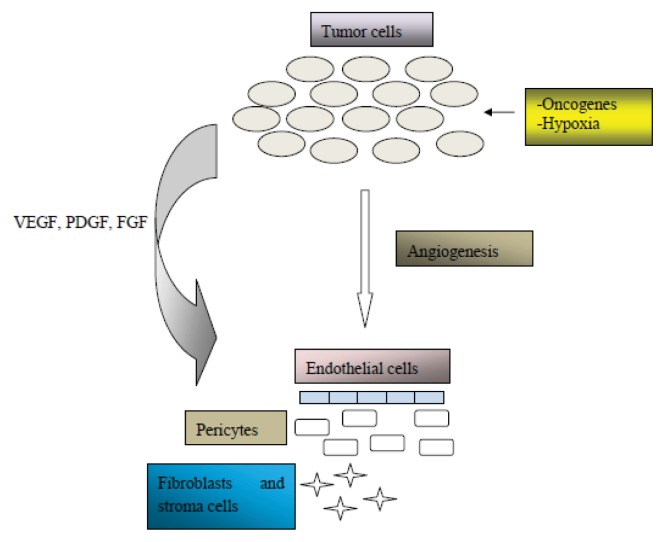
Angiogenesis activation from growth factors within the tumor microenvironment.

Pro-angiogenic factors’ action is counterbalanced by anti-angiogenic action by numerous other factors such as thrombospondin and angiostatin [40], and this balance has been termed the angiogenic switch [38]. In the case of normal tissues, the angiogenic switch is turned off, thus the vasculature remains quiescent as a result of the balance between pro- and anti-angiogenic factors [38,40]. In tumor tissues though, the exact opposite happens; balance is leaning towards the greater expression of pro-angiogenic factors and angiogenesis occurs [38]. These pro-angiogenic factors diffuse out of the tumor cells, during the course of tumor development, then bind onto adjacent endothelial cells in the case of mature blood vessels, thus triggering a process called vessel sprouting [41]. Sprouting seems to play a pivotal role in the angiogenesis process. When switching to the angiogenic phenotype according to the angiogenic switch process described earlier, the formation of new blood vessels occurs from pre-existing vasculature [42–45]. The newly formed vessels infiltrate the tumor mass in the local tumor microenvironment and promote tumor mass expansion, and subsequent hematogenous metastatic spread, therefore contributing to the pathogenesis of cancer. An overview of the angiogenic process is shown in Figure 1.

It may also be of importance to briefly mention that angiogenesis is involved in the metastasis of the tumor to the peritoneal cavity. At the time of metastasis, tumor cells from their organ of origin are secreted and move over to the peritoneum where they eventually reach the innermost layer of the peritoneum, the mesothelium. The mesothelium forms a cellular monolayer supported by a basement membrane. Tumor cells then adhere to the mesothelium, followed by penetration of the mesothelium so that tumor cells gain access to the submesothelial connective tissue. Invasion of the connective tissue provides the scaffold for further tumor proliferation, thereby establishing a metastatic deposit. The final step in this process is the induction of angiogenesis for sustainability of the tumor proliferation potential and also the achievement of further metastatic growth.

Peritoneal mesothelial cells have been shown to secrete angiogenic factors such as VEGF and Fibroblast Growth Factor (FGF) [46,47]. An increase in the secretion of such factors has been observed upon the stimulation with IL-1β and TNF-α, whereas factors such as IL-2 inhibited secretion of such pro-angiogenic proteins [46]. Such action for IL-1β has been shown to be possible in ovarian cancer as well, as a recent study has shown [48]. Gerber *et al.* have shown that the omentum was a major site of metastases growth for intraperitoneal tumors [49]. A subset of mesothelial cells located in the omentum was found to be hypoxic and also secrete VEGF. In addition, the presence of CD105+ vessels and localized sprouting indicated that active angiogenesis was occurring in the peritoneum.

In the case of ovarian cancer the seeding of the peritoneum and the resultant cancer development seem to be significant processes in the development of ovarian cancer and especially for the production of ascites [50]. The role of VEGF is quite important in this process. Experiments using animal models with peritoneal cancer spread have shown that VEGF overexpression may lead to tumor increase and also to peritoneal related neovasculogenesis and also increased vascular permeability in the peritoneum [51,52]. Therefore, there is a strong indication that angiogenesis may play a role in cancer metastasis in the peritoneum with mesothelial cells playing significant role in the process.

Angiogenic growth factors may also influence the growth of cancer cells *per se*. This has been shown by the direct effect of VEGF on to tumor cells [53]. It has also been shown that the autocrine VEGF/VEGFR loop, when both of these molecules are expressed in tumor cells, may be responsible for the growth enhancement of tumor cells *per se* [54]. The fact that VEGF plays a tumor cell proliferative role has also been shown in the case of ovarian tumor cells as well [55].

Angiogenesis plays an important role in all types of cancer, including gynecological [56–58], thereby a role for angiogenesis in the pathophysiology of all gynecological cancers including ovarian cancer has now been established. We hereby describe the important molecular pathways that are involved in this process in the pathogenesis and expansion of ovarian cancer.

## 3. Vascular Endothelial Growth Factor (VEGF) Related Pathways

VEGF plays an exceptional role in angiogenesis. It is involved in this process by mainly regulating new blood vessel growth [59]. It also promotes survival of immature vasculature before it turns into its mature form. VEGF was first discovered by Ferrara and colleagues [60] and was previously known as the vascular permeability factor due to its capacity of increasing vascular permeability [61]. There are seven member molecules that fall into this family of proteins, including VEGF A–E, and also the placental growth factor 1 and 2 (PIGF-1 and PIGF-2) [62,63]. VEGF-A multiple isoforms may be formed due to alternative mRNA splicing, with VEGF_165_ being the most prevalent VEGF isoform in a number of tumors [64–66].

Molecules of this family exert their effect via signaling through their tyrosine kinase receptor counterparts that are expressed normally on the surface of endothelial cells [62,64] and are termed vascular endothelial growth factor receptors (VEGFR). There are three isoforms of this type of receptors namely VEGFR1-3 [67–69]. VEGF-A binds preferentially to VEGFR1 and 2, VEGFB and PIGF-1 and PIGF-2 bind to VEGFR1, whereas VEGF-C and D bind preferentially to VEGFR3 [70,71]. The binding of the ligand onto the receptor induces a receptor dimerization that leads to intracellular signaling initiation.

VEGF expression has been shown to be upregulated by factors such as IGF-1 and IL-6 [72,73]. The expression of VEGF may also be regulated by mutations in genes such as *p53*, *ras*, *src*, and *vhl* [74,75] Function-wise, VEGFR2 is the main receptor isoform through which VEGF, mainly VEGF-A, mediates its effects that are directly related to angiogenesis [76,77]. VEGFR-1 has a less defined role, although recent studies have shown that both VEGF and PIGF may bind onto the receptor, in pathological conditions such as tumors, and enhance angiogenesis effects [78]. Moreover, soluble VEGFR1 may even play a role in controlling VEGFR2 signaling as it can act as a decoy receptor molecule [79]. In turn, VEGFR3 plays a lesser role in angiogenesis, but it has been documented to play an important role in lymphangiogenesis upon binding of VEGF-C and VEGF-D [59,80,81]. The main VEGF isoform being important in angiogenesis is VEGF-A and it will be referred as VEGF from this point onwards.

VEGF is produced by cancer cells and relates to the metastatic potential of a number of different types of tumors, including ovarian cancer [82,83]. It is detected by immunostaining in most ovarian cancerous tissues and it is also an important facilitator of the creation of ascites in the latter stages of the disease [84,85]. VEGF alongside its receptors constitute the dominant pathway that regulates angiogenesis in ovarian cancer [39,86]. The role of the VEGF/VEGFR axis in ovarian cancer has been well documented due to pharmacological studies of agents that reduce the burden of women with the disease [83,87].

Intracellular signaling related to VEGF in ovarian cancer includes the elaboration of molecules such as JAK and STAT pathway components, PI-3 kinases, and MAP kinases [82]. More specifically, PI-3K has been shown to play an important role in angiogenesis with its expression correlating with VEGF upregulation [88], and an upregulation of the PI3K/Akt pathway is observed [89]. The activation of the JAK-STAT pathway has been correlated with upregulation of VEGF and intracellular signaling in angiogenesis, especially the upregulation of STAT3 and STAT5 [90]. MAP kinases are also involved in an interplay with VEGF levels [91]. For the initiation of signaling, an autocrine loop of VEGF/VEGFR has been indicated to be responsible [36,40].

**Figure 2:**
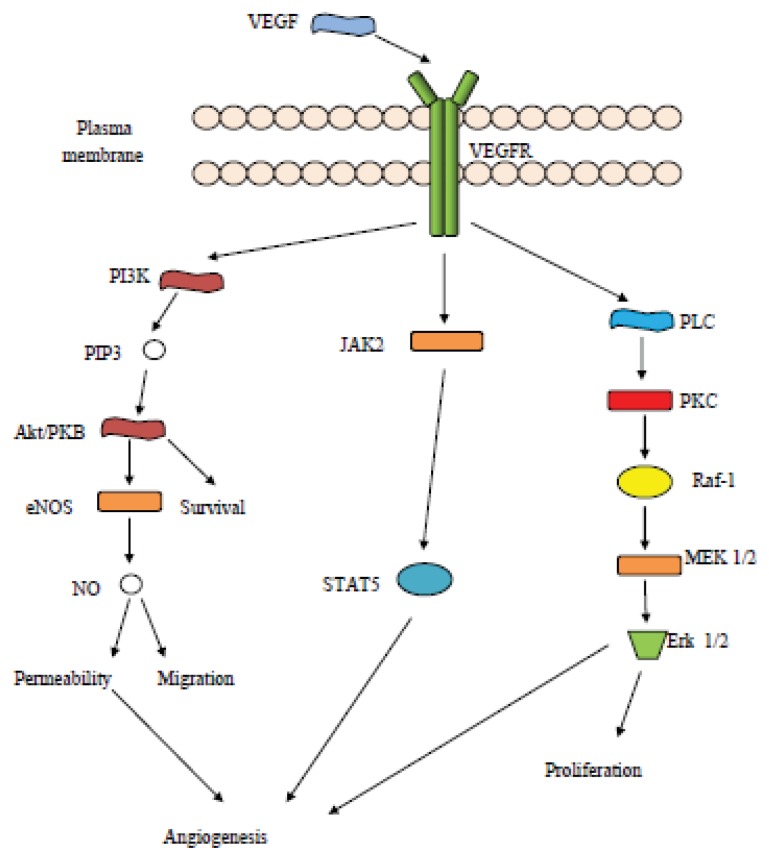
Schematic overview of the VEGF pathway.

Lately, there are other protein molecules that have been studied and shown to be involved in a signaling interplay with the VEGF/VEGFR complex, mainly VEGF/VEGFR2. These include the Src kinases, which increase vascular permeability [92], and phospholipase C that may interact with Erk/MAPK molecules enhancing the VEGF effect on vascular permeability and vessel formation [93]. Some elements of the VEGF pathway are shown in Figure 2.

## 4. The Platelet Derived Growth Factor (PDGF) Pathway

PDGF is an essential protein to pericyte recruitment, which is a critical aspect of blood vessel maturation. It has been shown that the activation of the PDGF Receptor (PDGFR) leads to upregulation of angiogenic events [94,95]. PDGF also interacts with VEGF and they either converge their signaling cascades or the PDGF pathway may be activated in response to resistance to VEGF inhibition [95,96]. The importance of PDGF in angiogenesis and in tumor progress is highlighted by the correlation of its expression with ovarian cancer patients’ prognosis [97].

Four isoforms of the PDGF molecule have been identified namely PDGF A-D [98,99]. As in the case of VEGF, there is specificity on which isoforms of PDGF bind specific corresponding receptor isoforms either PDGFR-α or PDGFR-β in order to exert their effects. In this case, PDGF A–C bind onto PDGFR-α, whereas PDGF-B and PDGF-D bind onto PDGFR-β [100]. As in the case of VEGF/VEGFR, an autocrine signaling mechanism, may be responsible for PDGF promoting angiogenesis and tumor growth [101].

Upon activation of the PDGF pathway, signaling occurs via the use of the PI3K/Akt complex pathway but there are also MAPK molecules involved alongside proteins of the Src family and Phospholipase C-γ [102]. Other molecules related to the PDGF signaling include the Ras protein [103], STAT proteins, and guanine-5′-triphosphate (GTP-ase) activating protein [104].

In the case of ovarian cancer, PDGF has been recorded in a large number of samples and a five to six-fold increase in the level of PDGF has been measured in ovarian cancer tumor cells when compared to cells of the normal ovarian epithelium [105–107]. PDGFR is expressed in ovarian carcinomas and it is also present in malignant ascites [108].

**Figure 3:**
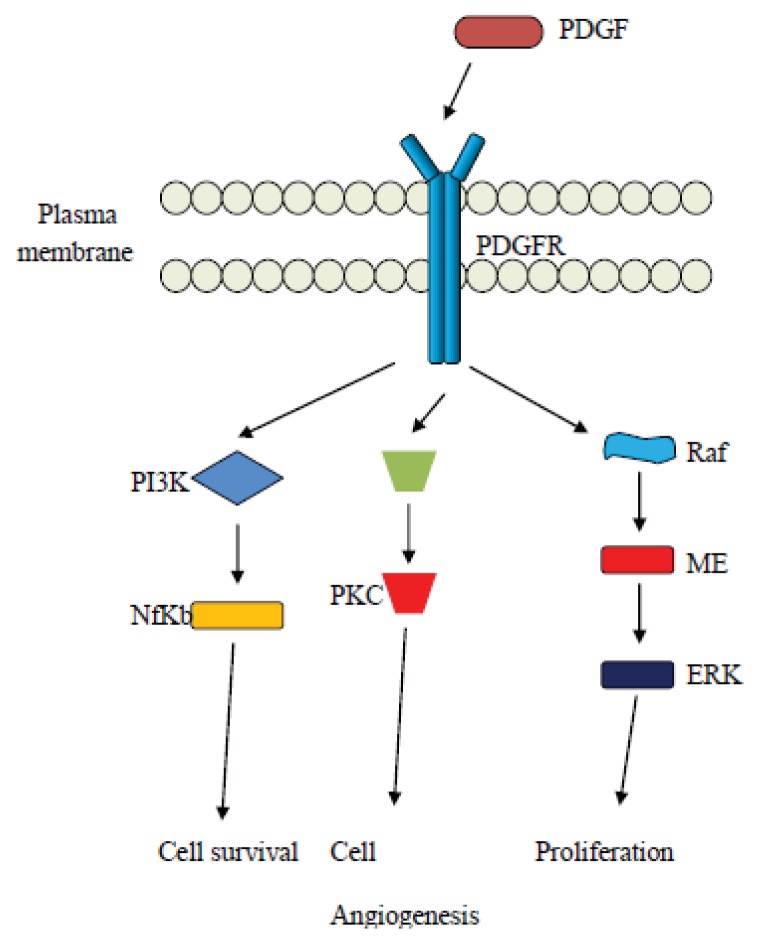
Schematic overview of the PDGF pathway.

PDGF has been shown to interfere with the stroma formation and also act as a substrate for angiogenesis [109]. It has also been shown to act in concert with VEGF in order to promote new vessel formation and stabilize newly synthesized vessels [75,110,111], so PDGF molecules are key regulatory molecules in oncogenesis and angiogenesis, important in ovarian cancer. Some elements of the PDGF signaling pathways are shown in Figure 3.

## 5. The Fibroblast Growth Factor (FGF) Pathway

FGF signaling mechanism has originally been studied as a significant embryogenesis pathway [112] and it has since become an important research target when it comes to angiogenesis research in cancer [113,114]. There are over 20 FGF isoforms identified, namely 23, and five receptor molecules (FGFR) have also been described [115]. The receptor molecules pose great similarity in structure, including an extracellular immunoglobulin (Ig)-like domain and an intracellular tyrosine kinase domain [116,117]. These domains are conserved between the first four isoforms of the receptor but the fifth isoform (FGFR-5) lacks the intracellular tyrosine kinase domain [118,119].

Upon binding of the ligand onto the receptor, the receptor molecules dimerise, a process that leads to the initiation of the intracellular signaling cascade. In ovarian cancer, disruptions to the appropriate signaling cascade have been reported, such as alternative splicing events differentiating the ability of the receptor to bind ligands effectively, while mutation events have not been considered significant in altering the receptor’s function [119–121].

In ovarian cancer, differences in alternative splicing may confer sensitivity to the ligand [122]. Moreover, in the case of ovarian cancer, FGF may be secreted into malignant ascites alongside VEGF, therefore, it may be contributing to cancer progression and angiogenesis [123,124]. The expression of FGF may be associated with prognosis [125].

It has been shown that FGF may play a direct role in tumor cell proliferation in ovarian cancer [126,127], but may also play a role in angiogenesis acting alongside other pro-angiogenic factors such as VEGF [128,129].

**Figure 4:**
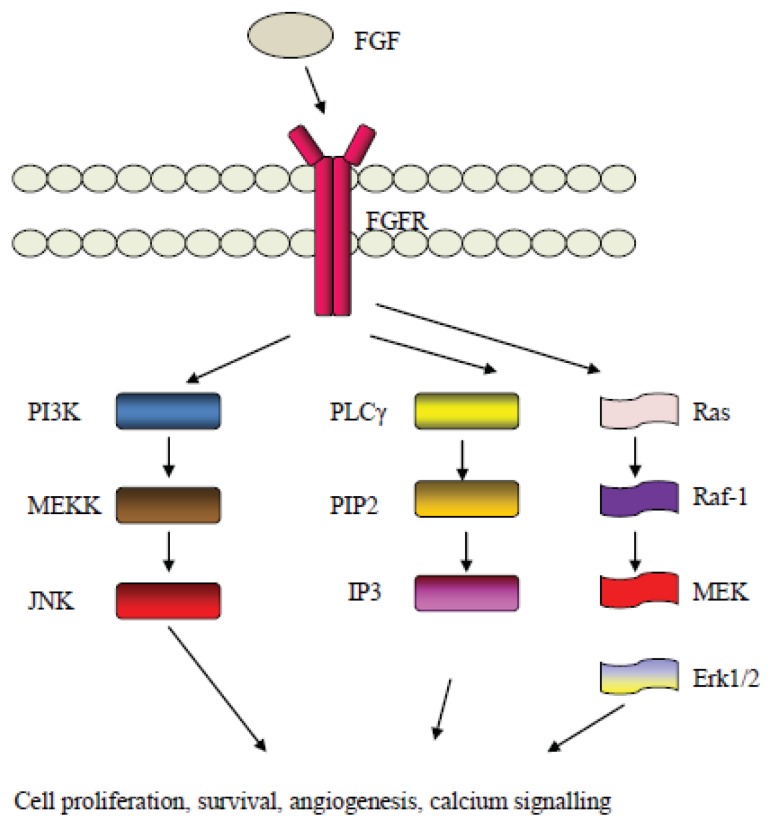
An overview of the FGF signaling pathway.

The FGF signaling pathway involves the employment of downstream proteins such as MAPK proteins and proteins of the PI3K/Akt cascade [130]. Phospsholipase-c and IP3 cascades are also involved in the downstream signaling of FGF, whereas the FGF pathway may crosstalk with other pathways such as the Notch pathway [131]. A schematic overview of some elements of the FGF related pathways are shown in Figure 4.

## 6. The Angiopoietin Pathway and the Tie2 Receptor

There are two forms of the angiopoietin (Ang) protein, namely angiopoietin-1 (Ang-1) and angiopoietin-2 (Ang-2), and both these proteins may interact with the Tie2 receptor [132]. Ang-1 and 2 may interact with Tie2 and enhance new vessel production [133], whereas Ang-1 acts via the use of the Akt/survivin pathway in order to stabilize newly produced vessels [134]. Ang-2 may act alone or in synergy with other pro-angiogenic factors, such as VEGF, in order to establish and enhance vasculature and it acts in promoting endothelial cell migration by blocking the vessel stabilizing action of angiopoietin 1 [135]. It has to be mentioned, though, that late studies show that Ang-2 may be acting in an agonist manner to Ang-1 when the latter is lacking or alternatively in a dose dependent manner when Ang-1 is actually present [136]. Although in the normal ovary, Tie2 localization may indicate communication between the extracellular matrix and the endothelial cells, in mouse ovarian tumor models Ang-2 is mainly expressed in endothelial cells and the tumor stroma and its expression levels correlate with those of VEGF, therefore proposing a synergistic effect of the two molecules in ovarian cancer angiogenesis [137].

**Figure 5:**
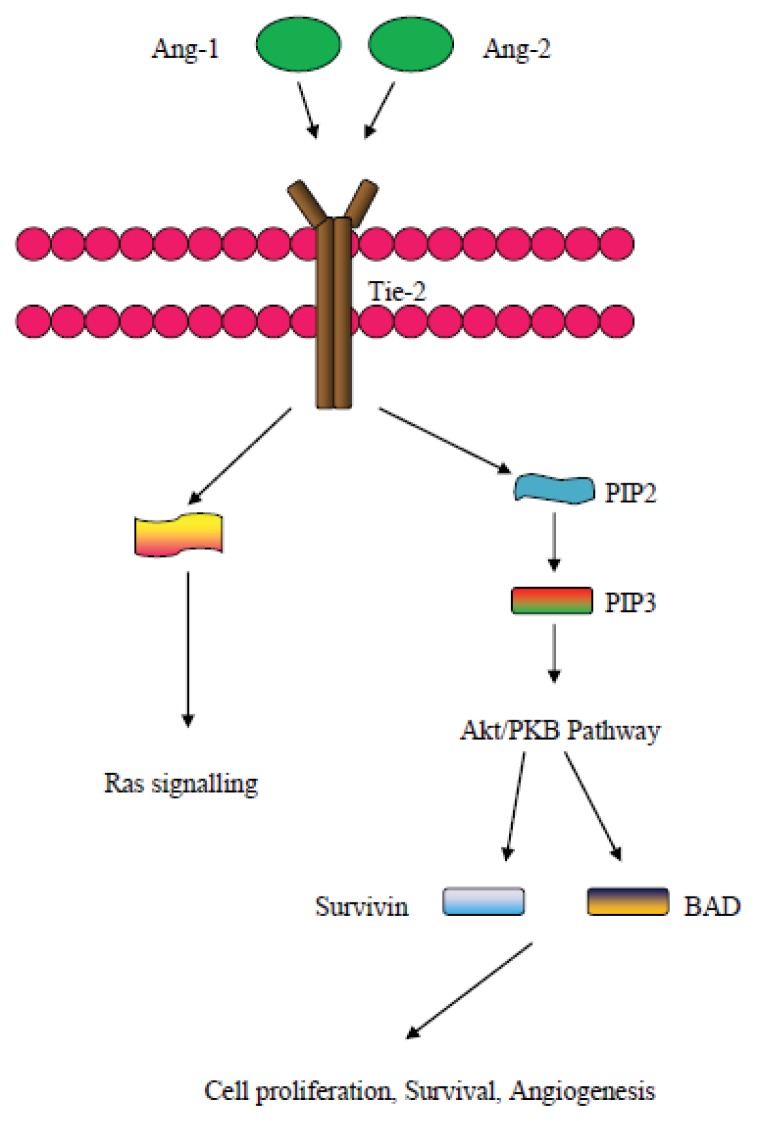
An overview of the Ang signaling pathway.

Other molecules involved in the downstream signaling pathway of the Ang/Tie2 receptor include the PI3K protein, involved in the PI3K/Akt pathway, and also proteins such as Protein Kinase B, MAPK/Erk molecules, and also molecules of the Ras pathway [135,138], and these molecules may possibly, also, be involved in the downstream signaling of the Tie2 receptor in ovarian cancer. Finally, recent studies show that Ang-2 may be exerting its signaling effects via the employment of integrin molecules [139]. The possible effects of Ang in angiogenesis can be further exhibited by the blocking of the binding of Ang onto the Tie2 receptor that leads to decreased sprouting and reduction of the number of tumor vessels [138]. Figure 5 depicts some of the signaling elements of the Ang related pathways.

## 7. Targeting the Angiogenesis Related Pathways for Ovarian Cancer Treatment

Since angiogenesis poses an important process for ovarian cancer dissemination it is of significance to attempt to devise therapeutic strategies that target angiogenesis pathways. So far strategies that target molecules such as VEGF and PDGF have been developed that act alone or in combination with chemotherapy in order to achieve a more effective treatment [75,82].

Anti-angiogenic agents include bevacizumab, an anti-VEGF monoclonal antibody that has exhibited satisfactory action as a single-phase treatment agent in phase II trials in recurrent epithelial ovarian cancer [75,87]. Bevacizumab is a humanized monoclonal antibody that binds onto VEGF (mainly VEGF-A) with high affinity, thus neutralizing the VEGF activity. Bevacizumab has also been used in combination with other therapeutic agents such as platinum compounds e.g. carboplatin, and also paclitaxel, nab-paclitaxel, topotecan, doxorubicin, and docetaxel [140–144]. In terms of pre-clinical studies Mesiano *et al.* tested Bevacizumab;s activity in immunodeficient mice and showed that the drug inhibited subcutaneous tumor growth, partially inhibited the tumor’s intraperitoneal growth, and completely inhibited ascites formation [145]. In another study the synergistic effect of bevacizumab with paclitaxel has been described reducing tumor growth and ascites formation [146]. Mabuchi *et al.* also showed that the continuous administration of bevacizumab could significantly prolong survival *in vivo* [147]. Recent results established the role of Bevacizumab in ovarian cancer, showing that it increases the efficacy of chemotherapy in the initial management of the disease [27,148] but also in relapsed platinum-sensitive [29] and platinum-resistant [149] disease.

Another anti-angiogenic agent is aflibercept or VEGF-Trap as it is commonly called, which is a fusion protein combined from the domain 2 of VEGFR1 with domain 3 from VEGFR2, attached to the hinge region Fc of a human IgG1 [150]. VEGF-Trap binds all isoforms of VEGF and confers a neutralizing effect [150,151]. Research is ongoing concerning the efficacy of the agent. Pre-clinical data on aflibercept has shown that it is able to inhibit the angiogenic effect including narrowing of vessels, endothelial cell apoptosis, and stop of blood flow and also reduction of tumor burden and ascite formation [152,153]. VEGF-Trap has also been used in combination with other therapeuting agents such as docetaxel and cisplatin [154,155]. Preliminary results from a phase 1/phase 2 trial of aflibercept in combination with docetaxel in patients with recurrent gynecologic malignancies, including ovarian cancer, reported promising preliminary findings [155].

Except the anti-VEGF inhibitor molecules, multiple anti-VEGFR inhibitor molecules, termed anti-angiogenic tyrosine kinase inhibitors are currently undergoing investigation. BIBF 1120 (Intedanib) is an agent that blocks the activity of VEGFR 1–3, PDGFRa and PDGFRb, and FGFRs [156]. Pre-clinical data show that BIBF 1120 exhibits high activity in decreasing vessel density and reducing tumor growth in mouse models [157]. BIBF 1120 has been used as a single agent, but also in combination with the combination of carboplatin/paclitaxel has also been used in epithelial ovarian cancer patients [158].

Pazopanib is another tyrosine kinase inhibitor that inhibits the activity of VEGFR 1–3 and also PDGFRa and PDGFRb, and FGFR-1 and FGFR-3 [159]. Pre-clinical data showed that Pazopanib may inhibit VEGF and FGF induced angiogenesis in mouse models, although the effect was higher related to VEGF stimulation [160]. Studies using Pazopanib as a single agent are currently undergoing [159]. Cediranib consists another multiple tyrosine kinase inhibitor, thus neutralizing the effect of molecules such as VEGFR 1–3, FGFR-1, and PDGFRa and PDGFRb [161,162]. In the case of cediranib pre-clinical data has shown that the drug inhibits angiogenesis in ovarian cancer in a dose dependent manner [163]. Clinical trials in phase II are currently underway and there is also a necessity for trials including a therapeutic combination of cediranib and platinum therapeutic agents.

Sorafenib is a tyrosine kinase inhibitor that neutralizes the effect of VEGFR-2, VEGFR-3, and PDGFRb [164]. Studies are ongoing and results for some of them are currently in process [164]. Pre-clinical data have shown the drug to inhibit tumor growth in nude mice and reduce tumor growth at a significant level [165]. Sunitinib inhibits VEGFR-2 and PDGFRB among other molecules. Sunitinib related pre-clinical data have shown also that it can inhibit tumor growth and reduce microvessel density count [166]. Some studies exhibit modest efficacy results [167] but other studies are still ongoing.

Finally, AMG-386, is a fusion protein that inhibits the binding of both Ang-1 and Ang-2, and Tie2 receptors [168]. Pre-clinical data shows that AMG-386 is directly involved with intracellular signaling as studies in mouse models have exhibited [169]. Results from studies using AMG-386 as a single agent or in combination with other agents, such as bevacizumab, are awaited [170].

Separate reference should be made to the hypothesized antioangiogenic effect of paclitaxel. Paclitaxel is used alone, or in combination with other anti-angiogenic agents such as Bevacizumab. The actual mechanism of its anti-angiogenic effect is not clear yet. A few hypotheses on that matter have accounted for that effect, including the inhibition of endothelial cell morphogenesis *in vitro* [171]. In another study paclitaxel seems to have an antiangiogenic effect due to a possible increased uptake by endothelial cells [172]. We should also note here that paclitaxel has been shown to increase *Cox-2* mRNA expression, which is a pro-angiogenesis molecule, and thus a combined treatment of paclitaxel alongside a *Cox-2* inhibitor molecule may be desirable in some cases [172].

A number of studies have also attempted to shed more light in predicting the effect of anti-angiogenic drugs, mainly via the usage of potential biomarkers that may be used for this purpose. Such examples include the possible use of VEGF as a biomarker when attempting to predict the effect of bevacizumab [173,174], with different isoforms varying in their reliability as predictive factors of the function of the drug. PIGF is another angiogenesis related molecule that may act as a biomarker for predicting the effect of anti-angiogenic drugs as its levels have been shown to be elevated upon usage of almost all anti-VEGF drugs [175]. In the same way, soluble fragments of VEGF receptors may play such roles for predicting the effects of cancer anti-angiogenic agents. Examples include the soluble form of VEGFR-1 (sVEGFR1), where the high plasma concentrations of the molecule in blood circulation may predict a poor outcome for patients treated with agents such as bevacizumab and sunitinib [176].

Finally, other molecules that may play a role in assisting prediction of the effect of anti-angiogenic drugs include collagen IV in the case of using a number of angiogenic agents [177], and IL-8 in the case of using sunitinib [178].

Antioangiogenic therapy, though, has not been so far able, though, to completely cure the disease due to the fact that resistance develops during this type of tumor treatment. Postulated mechanisms for overcoming anti-angiogenic therapy include the use of an alternative pathway when one is blocked [179], hypoxia by accounting for the selection of more aggressive cells [180], and also by assisting the survival of cancer stem-like cells [181]. Other causes for resistance include the recruitment of vascular progenitors and modulators such as the pericyte progenitor cells [182], and tumor cell dormancy that tumor cells enter upon the effect of different types of cell stress [183].

## 8. Conclusions

Angiogenesis represents a hallmark process that leads to cancer dissemination. Pathways that are related to angiogenesis consist of biochemical processes that occur downstream of the binding of molecules such as VEGF, PDGF, FGF, and Ang with their cognate receptors. Through studying these pathways, molecular targets for developing therapeutic strategies have emerged. Anti-angiogenic molecules have been developed and many clinical trials are underway. It is of importance for angiogenesis research in ovarian cancer to continue, since this is a promising area for devising more effective treatments against gynecological cancer.
